# Supplementary material for: Are carbon emissions trading and green financial instruments synergistic? -Comprehensive quantitative research based on content analysis
Source: PLoS One. 2024 Mar 7;19(3):e0298601. doi: 10.1371/journal.pone.0298601 (PMC10919601; doi:10.1371/journal.pone.0298601)
Supplement: S1 Data — (ZIP) [file pone.0298601.s001.zip › Data/地级市Z_F工作报告.zip!/┴╔─■/░░╔╜╩╨/└┤╫╘╣1⁄2╓┌║┼í╛┬φ┐╦╩2╛▌═°í┐_2017.docx]

各位代表：

现在，我代表市政府向大会报告工作，请予审议，并请市政协各位委员提出意见和建议。

一、2016年工作回顾

过去一年，是鞍山发展进程中极不平凡的一年。面对前所未有的经济下行压力，面对前所未有的矛盾风险挑战，我们在省委、省政府和市委的坚强领导下，在市人大、市政协的监督支持下，认真学习贯彻习近平总书记系列重要讲话精神，坚持稳中求进工作总基调，全面落实五大发展理念和“四个着力”，积极推进供给侧结构性改革，保持定力，攻坚克难，爬坡过坎，负重前行，全市经济运行逐步趋稳，质量效益逐步提升，发展态势逐步向好，多项重点工作走在全省前列，为全面振兴钢都奠定了扎实的基础。

“逐步趋稳”主要表现在，钢铁等主导产业快速恢复，预计生铁、粗钢、钢材产量分别增长7.3%、9.1%和11.8%，规模以上工业企业产销率达到99.4%。工业品出厂价格指数从8月份开始结束了连续33个月的负增长，持续保持正增长。就业形势总体稳定，全年城镇新增就业4.3万人，城镇登记失业率控制在2.9%以内；城镇和农村常住居民人均可支配收入分别增长5.5%和7.5%。

“逐步提升”主要表现在，经济运行效益明显好于上年，一般公共预算收入增长3%，非税收入占比降至近年来较好水平。工业增值税增长10.3%。服务业增加值占地区生产总值比重达到49%，比上年提高2.4个百分点。

“逐步向好”主要表现在，经济运行先行指标呈现良好增长态势，工业用电量从3月份至今始终保持当月正增长，8月份开始一直保持在全省前三位，12月当月增长20.5%，全年增长8.4%。货物周转量增长3.1%。金融机构本外币贷款余额比年初增加172.7亿元。

“走在前列”主要表现在，我们全面完成了省重点考核的各项工作任务。上解养老保险风险基金、化解钢铁产能、取缔10吨及以下燃煤锅炉、去房地产库存、棚户区改造和行政审批制度改革、投资体制改革、商事制度改革、农村综合改革、医药卫生体制改革等多项工作居于全省前列。

各位代表，一年来，在困难超出预期、压力超乎寻常的情况下，我们始终保持发展定力，坚定发展自信，脚踏实地，埋头苦干，直面问题，应对挑战，重点抓了以下工作：

（一）抓经济平稳运行

着力稳定实体经济。开展困难企业帮扶工作，坚持“一企一策、精准帮扶”，实施档案化管理和包保责任制。累计为284户企业解决历史遗留问题351个，为763家企业补办环评审批手续，使企业卸下历史包袱。一次性为鞍钢矿业集团5802公顷土地办理土地证，解决了困扰鞍钢十余年的周边矿山土地无证问题，增加鞍钢资产208.5亿元。落实企业减负政策，减免企业税费6.13亿元，清理涉企收费1.56亿元，节省企业购电成本8300万元。全年新增升规入库工业企业66户。开展促进消费扩大升级行动，大力发展会展业、“夜经济”和节日经济，社会消费品零售总额增长5%。商品房销售面积317万平方米，增长5%，库存比上年底减少29.2万平方米。进一步做实对外开放的基础性工作，制定出台促进进出口相关政策措施，积极与军贸企业对接，培育鞍山紫竹等综合性外贸服务企业，实现了本地外贸服务企业零的突破。西柳现代物流园及海关监管区开工建设。辽宁凯鹏等企业建立了海外仓。海城农机物流园等6个物流园列入“一带一路”战略节点项目。

着力推进重点项目建设。海城瑞丰项目开工建设，缘泰化工项目试生产，辽宁衡业汽车新材高性能钢板热处理等一批重大项目投产达产。全年开工建设亿元以上项目412个，其中新开工项目107个。组织各类招商100余次，签约5000万元以上项目275个。依法解决了一批多年遗留的拆迁难题，市本级完成腾迁净地57宗、378.8公顷，盘活闲置厂房16.9万平方米，使一批重点项目具备开工条件。

着力破解融资难题。累计争取各类资金239亿元，其中争取中央专项建设基金43.9亿元，争取中央预算内和上级补助资金9.95亿元。融资总量达到720亿元。争取国开行贷款余额65亿元。全市33家企业分别在“新三板”及区域性股权交易市场挂牌融资。鞍钢集团成功发行钢铁行业首只私募公司债，海城金财发行企业债券10亿元。成功签约PPP项目3个，融资21.8亿元。

（二）抓重点领域改革

协调推进“放管服”改革。新取消行政审批事项14项，清理中介服务事项12项，审批要件减少20%。简化优化公共服务流程，为基层群众办事创业提供了更加高效便捷的服务。成立行政审批服务局，实行“两集中两到位”改革，全市32家单位实现行政审批事项进大厅。建成投资项目在线审批监管平台，全面推行“双随机一公开”监管。出台优化营商环境的实施意见，成立优化营商环境监督办公室，设立企业投诉热线，对重点单位开展专项巡查，实现了营商环境常态化监管。

加快推进国企和经营性事业单位改革。整合国有资源，组建交通运输、建设投资等9家企业集团，完成注入资产78.8亿元，引进资金11.4亿元。全面完成供销社改革，历史遗留问题得到彻底解决。对鞍山一工、合成集团等企业实施了资产重整。推进驻鞍央企“三供一业”分离移交工作。出台生产经营性事业单位改革方案。置换政府债务250亿元，减少利息支出10亿元，债务风险总体可控。

大力支持民营经济发展。出台加快发展民营经济实施方案，获批东北地区民营经济发展改革试点。建设中小微企业信用体系，实行小微企业“一照多址”备案登记办法，持续推进“五证合一”“一照一码”“两证整合”登记新模式和“先照后证”改革。全年新设立市场主体3.2万户。

积极推进农村各项改革。完成120万亩土地确权登记颁证工作。农村产权交易总金额达到5.2亿元。海城市国家农村改革试点工作取得重大突破，成立了农村土地承包经营权流转中心，建立了东北首家农村综合产权交易平台和监管平台，获批全省农村产业融合示范区。

（三）抓产业转型升级

促进工业提质增效。出台了工业供给侧结构性改革实施意见，鞍钢与神户制钢合资60万吨高强汽车板、紫竹集团年产100万吨城市轨道钢等一批钢铁深加工项目建成投产，海鸣矿业等一批菱镁新材料项目顺利推进。新认定高新技术企业30家。激光科技产业集群工业增加值增长45%，森远集团、聚龙股份等民营企业实现由生产型制造向服务型制造跨越。支持鞍钢发展，与鞍钢集团签订“智慧城市”建设战略合作协议，煤焦油深加工产业园等合作项目取得实质性进展。压减钢铁产能160万吨。

加快服务业提档升级。西柳物流园被商务部确定为智慧物流配送示范基地，与中国供销集团签订农产品流通体系建设战略合作协议。新建各类专业市场12个，德邻物流港启动运营，苏宁生活广场等8个商业项目竣工，新增金融机构4户，电子商务交易额增长2.7倍。整合千山风景区和玉佛山风景区旅游资源，组建辽宁千山旅游集团。积极推进千山5A级景区创建工作，新增3A级景区14家，岫岩进入国家全域旅游示范区创建名单，御汤泉欢乐水世界等7个重点旅游项目建成营业，旅游总收入增长15%。

大力发展县域经济。出台了农业供给侧结构性改革实施意见，调减籽粒玉米面积13.7万亩，设施农业总面积发展到50万亩，粮食产量达到147万吨。开工建设农业项目28个，其中亿元以上项目4个，规模以上农产品深加工企业发展到181家。辽宁义乌中国小商品城一期投入运营，后英特种镁砂项目建成试生产，安井食品二期工程建成投产。

（四）抓科技创新驱动

加大科技创新扶持力度。出台鼓励创新优惠政策。制定《中国制造2025鞍山行动纲要》，鞍钢智慧矿山建设列入国家钢铁工业调整升级规划。鞍钢矿业、荣信股份技术研究项目入选国家重点研发计划，53个企业技术创新项目列入省重点计划，22个产品通过省中小企业“专精特新”产品技术认证。

积极推进“双创”工作。市政府设立产业创业投资引导基金，组建了5亿元激光产业股权投资基金和科创高端装备创业投资基金，6户企业获得省创业引导基金2.4亿元。建成鞍山科技大市场，新增众创空间等孵化平台3家，创新创业示范基地达到32个，在孵企业、团体1138个，实现了每个县（市）区拥有一个创新创业示范基地目标。全市完成技术合同交易额10亿元，发明专利申请量1355件，聚龙股份等3家企业获得年度市长质量奖。

深化政产学研合作。成功举办“院士专家鞍山行”活动，新增院士专家工作站3个。新增滑石新材料等3个产业技术创新战略联盟。5个项目列入国家火炬计划。辽宁科技大学搭建校企协同科技创新平台，鞍山师范学院打造创新创业教育改革新模式。

（五）抓保障改善民生

在财政收支矛盾突出的情况下，坚持保基本、兜底线、促公平，民生投入增长5.6%。

加大脱贫攻坚力度。出台扶贫脱贫优惠政策，市本级投入扶贫资金7313万元，落实扶贫信贷风险基金2000万元，新增扶贫贷款6055万元。完成贫困户危房改造1118户、易地搬迁改造127户，实施黑色路面工程354公里，对困难家庭学生每人增加1000元补助，对贫困人口实施了大病保险。全市9120名贫困人口实现脱贫，28个贫困村销号。

全面完成回迁房建设。回迁房建设是社会关注的焦点、百姓关心的热点、民生工作的难点。我们把回迁房建设作为民生“一号工程”，签下军令状，压实责任，挂牌督战，逐一解决主体工程建设及供电、供水、供气和小区环境等方面难题，建成回迁房项目18个，建筑面积256万平方米，回迁居民2.8万户、近10万人，实现了2014年以前动迁户全部回迁安置，多年的民生难题终于得到有效化解。

不断完善社会保障体系。着力解决企业养老金缺口，确保了企业养老金按时足额发放。有效解决了“五险合一”老大难问题，实现医保省内异地就医联网直接结算。城市低保标准提高5%，农村低保标准提高10%，农村五保对象保障标准提高7%，为4.1万户城乡低保家庭实施救助。深化户籍制度改革，为3473名无户籍人员办理了登记落户。完成1.8万户棚户区改造任务。

着力打造百姓宜居环境。全国文明城市创建工作取得新进展。完成玉佛山绿道示范段建设、千山中路改造、人民路大修等基础设施项目，建成客运站过街天桥、二一九公园正门地下通道，全面完成以烟草岗为重点的道路交通拥堵整治工程，开通市区至唐家房公交线路。实施了城市亮化美化工程，城市脏乱差问题进一步解决。推进智慧城市建设，启动智能公交、智能医疗、智能城管等项目。新采购新能源公交车400辆，开通鞍山至成都航线。启动城市老旧小区改造工程182万平方米。完成城市供水管网改造，市区近5万户、15万居民结束了定时供水的历史。新建换热站24座，新建改造供热管网180公里，进一步提升了供暖质量。完成农村危房改造3130户。创建宜居示范镇10个、宜居示范村60个，台安县桑林镇成为国家新型城镇化综合试点镇。深入推进青山、碧水、蓝天、沃土四大工程，完成蓝天工程项目16项，取缔10吨及以下燃煤锅炉184台，完成省下达指标的175%；淘汰黄标车和老旧车辆35692辆，完成省下达指标的172%。全年空气质量达标天数290天，达标率79.2%，比上年提升15.2个百分点，空气质量达到历史最好水平，改善幅度全省排名第一。

大力发展社会事业。获批国家职业教育“双元”试点，改造农村薄弱学校20所。“三医联动”改革取得积极成效，公立医院药占比下降4%，节约药品采购成本5000余万元。市公共卫生中心全面建成，解决了长达7年之久的历史遗留问题。成功举办2016年全国乒乓球锦标赛和首届鞍山文化艺术节。新建、改扩建城乡社区服务设施104个，科技馆、档案馆、青少年活动中心、妇女儿童活动中心等公共文化设施建设全面启动，高新区广场恢复工程依法推进。着力建设“学雷锋志愿服务城”，鞍山“发明之家”王春岩家庭荣获“全国文明家庭”称号，蝉联全国双拥模范城六连冠，荣获全国人民防空先进市，获批国家社会信用体系建设示范市。加强立体化社会治安防控体系建设，着力抓好安全生产、食品药品安全监管和信访维稳等工作，社会大局保持和谐稳定。

广播电视、新闻出版、妇女儿童、老龄、残联、民族、宗教、外事、侨务、对台、统计、接待、档案、编制、气象、地震、民兵预备役等工作取得了新的成绩。

（六）抓政府自身建设

严格落实党风廉政建设责任制，着力建设廉洁政府。坚持把全面从严治党要求落实到政府工作全过程、全领域，认真贯彻《中国共产党党组工作条例》，深入开展“两学一做”学习教育，扎实推进中央巡视“回头看”反馈意见整改落实工作，基本完成公车改革任务。严格落实中央八项规定精神，加强党风廉政建设，着力构建干部清正、政府清廉、政治清明的从政环境。

大力推进民主法治建设，着力建设法治政府。全年办理人大代表建议和政协提案522件，见面率、办复率均为100%。制定《关于加快建设法治政府的若干意见》和《市政府重大行政决策程序规定》，完善政府法律顾问制度。制定和修改政府规章9件，市政府直接受理行政复议案件30件，依法行政能力进一步提升。

强力推进各项工作落实，着力建设诚信务实政府。制定《2016年政府工作任务及责任清单》，坚持市政府领导班子周例会制度，以每周为节点推进工作落实。全面落实市十二次党代会精神，精心组织研究和制定全面振兴钢都首批“1+13”重大专项推进方案。积极兑现政府承诺，妥善解决了诸多历史遗留问题。顶着巨大的经济下行压力，坚持实事求是，主动挤压水分，做实经济数据，为今后经济健康发展打下了良好基础。加大督查力度，督办事项501件（次），使认真较真抓落实成为工作常态。

回顾一年来的政府工作，我们深刻体会到：

必须牢固树立“四个意识”，特别是核心意识、看齐意识，坚决维护以习近平同志为核心的党中央权威和集中统一领导，坚定不移贯彻落实中央和省市委的决策部署，确保政令畅通、执行有力、落地生根。

必须持续改革创新，敢涉险滩，敢啃硬骨头，大胆探索，先行先试，推动改革向纵深发展，实现重点突破，追求高质量、高效益发展。

必须强化责任担当，坚持依法依规办事，打造法治诚信政府，做到新官理旧账，遇到矛盾不回避、碰到困难不推诿，不把风险推给上级，不人为制造新的风险。

必须做到爱民为民，始终把老百姓的事情放在心上，把造福百姓的责任扛在肩上，把百姓的满意作为工作的最高标准，让百姓多得实惠、生活得更好。

必须坚持求真务实，说老实话、干老实事，不做虚功，不图虚名，不搞“忽悠”，坚持说了就算、马上就办、办就办好，一步一个脚印抓好工作落实，在苦干实干中破解难题、推动发展。

各位代表，过去一年，我们迎难而上、负重前行，全市经济社会发展保持总体平稳。这是市委正确领导和市人大、市政协鼎力支持的结果，是全市人民凝心聚力、不懈奋斗的结果。在此，我代表市政府向全市人民，向各位人大代表、政协委员和各民主党派、工商联、无党派人士、人民团体，向鞍钢等中省直单位、驻鞍部队、武警官兵，向社会各界人士和离退休老同志，向所有关心支持鞍山发展的海内外朋友们，表示衷心的感谢和崇高的敬意！

各位代表，我们清醒地认识到，按照振兴发展的新要求、人民群众的新期盼，工作中还存在一些突出问题。主要表现在：一是受经济下行压力加大和做实经济数据双重影响，地区生产总值、固定资产投资、规模以上工业增加值等指标下降幅度较大，没有完成年初预期目标。二是社会投资意愿下降，企业信心不足，重大项目较少，经济发展后劲不足。三是创新创业氛围不浓，自主创新能力不强，新兴产业规模较小，产业转型升级步伐不快，城市竞争力和影响力不足。四是体制机制障碍依然突出，营商环境需要进一步改善。一些干部不会作为、不敢作为、工作不在状态的现象仍然存在。对于这些问题，我们将在今后工作中认真加以解决。

二、2017年工作安排

今年是实施“十三五”规划的关键之年，是供给侧结构性改革的深化之年，也是落实省、市十二次党代会精神的开局之年。当前，鞍山面临的发展机遇前所未有，面临的风险挑战前所未有。我们必须肩负起全面振兴钢都的重任，举全市之力，聚全民之智，抓住机遇，迎难而上，坚决完成时代赋予的光荣而艰巨的历史使命！

今年政府工作的总体思路是：以习近平总书记系列重要讲话精神为统领，全面贯彻党的十八大、十八届三中、四中、五中、六中全会和中央、省、市经济工作会议精神，紧紧围绕省、市十二次党代会确定的目标任务，按照“五位一体”总体布局和“四个全面”战略布局，坚持稳中求进工作总基调，坚定不移落实新发展理念和“四个着力”，主动适应引领经济发展新常态，牢牢抓住新一轮东北振兴重大机遇，以提高发展质量和效益为中心，以推进供给侧结构性改革为主线，以振兴实体经济为重点，深入实施“四个驱动”，培育壮大“六个新增长点”，抓牢抓实“一条主线、三大任务、四个目标”，全面做好稳增长、促改革、调结构、惠民生、防风险各项工作，促进经济平稳健康发展和社会和谐稳定，奋力开创全面振兴钢都新局面。

今年全市经济社会发展主要预期目标是：地区生产总值增长6.5%，固定资产投资增长12%，社会消费品零售总额增长9%，一般公共预算收入增长5%，规模以上工业增加值增长7%，城镇登记失业率控制在3.7%以内，城乡居民收入增长与经济发展同步。

在新的一年里，重点要做好以下工作：

（一）抢抓重大机遇，构筑发展新优势。紧紧抓住中央支持东北振兴的重大政策机遇期，持之以恒向上争、对外招、拼命干，不断夯实全面振兴基础，推动经济企稳向好。

认真落实国家政策。按照国发62号文件和国办发82号文件确定的重点支持政策，全力争取、主动配合、精准对接、逐项落实，力争将鞍山市列入国家产业转型升级、国家新型智慧城市等一批国家重要示范试点，努力在深化改革、创新转型、扩大开放等各个领域实现新突破，让政策支持效应在鞍山得到充分释放。

全力实施重大项目。着力推进纳入中央和省重点项目库的重大项目建设，逐个项目落实开工前期要件，最大限度保证项目前期经费支持，确保一批重点项目竣工投产。重点推动鞍钢集团、五矿集团等央企投资项目落地建设，实施鞍山机场改扩建、沈海高速汤岗子互通立交等一批重大基础设施项目，推进老工业区和独立工矿区搬迁改造。把招商引资作为促进钢都振兴的重中之重，举全市之力破解招商难题。紧盯重点区域，突出抓好利用闲置厂房和帮助停产半停产企业兼并重组、引进合作伙伴等方式进行招商。全年开工亿元以上项目369个，其中新开工项目158个。

积极筹措发展资金。围绕中央预算内投资重点投向，加大争取资金力度。建立专项建设基金项目储备库，设立鞍山市产业转型升级基金、鞍钢产业转型发展基金、新材料和高端装备等创投基金，实现与国家基金全面对接。落实促进民间投资健康发展政策措施，进一步拓宽民间资本投资渠道。在扩大银行信贷资金的同时，积极拓展债券融资、资本市场融资等直接融资渠道，吸引保险、信托等资金，推进企业上市融资。

（二）完善体制机制，释放发展新活力。发挥改革的引领作用，大力促进投资便利化、服务智能化、贸易自由化，不断激发内生发展动力。

持续优化营商环境。认真落实《辽宁省优化营商环境条例》，开展营商环境建设年活动，扎实推进东北地区民营经济发展改革试点和创建国家社会信用体系示范城市建设工作，实施规范行政审批和行政执法、规范中介服务、落实政府承诺等专项行动，扎实做好困难企业帮扶工作，保护各类市场主体合法权益，激发民营经济的活力和创造力，着力构建“亲”、“清”新型政商关系。

深化“放管服”改革。推进市对县（市）区管理改革，进一步下放权力，实现事权、管理权、服务权和财权有效划分。深化行政审批“两集中两到位”改革，强化审批和服务项目标准化，加大网上审批力度。推进投资和服务便利化改革，试行企业投资项目承诺制，在高新区、经开区先行开展“零审批”试点。继续深化商事制度改革，做好市场准入负面清单制度试点市工作。

加大国企和经营性事业单位改革力度。以国有企业混合所有制改革为突破口，引进战略投资者，做大做强市属国有企业。支持鞍钢等央企解决历史遗留问题，完成“三供一业”分离移交工作，积极稳妥推进厂办大集体改革。推进生产经营性事业单位转企改制。

深化农村各项改革。扎实推进海城国家农村改革试点，尽快形成可复制可推广的经验。稳步推进农村集体产权制度改革，开展集体资产清产核资，健全台账管理制度，做好村级集体经济组织股份合作社试点工作。推进土地确权登记颁证工作，鼓励适度规模经营，支持农民开展专业合作和股份合作。促进农村金融改革，建设抵押担保服务平台。

实施更加积极主动的开放战略。全面落实“一带一路”战略，支持鞍钢、紫竹等企业组建“走出去”战略合作联盟，建设海外工业园区。主动接受辽宁自贸试验区辐射，规划建设国际产业合作区。打通物流大通道，推进德邻物流园二期、西柳国际物流园等项目建设，建设海关商检监管场所，争取成为国家级市场采购贸易试点。积极参与区域通关一体化改革，建设现代通关体系。实施对口合作，学习江苏等地先进经验，开展干部人才交流培训、产业协作和重点区域合作。规划建设鞍山出口加工基地，承接加工贸易梯度转移。

（三）促进结构调整，构建产业新体系。坚持质量引领，提高供给体系的质量效益和全要素生产率，推动产业迈向中高端，全面增强实体经济竞争力与活力。

深入推进“三去一降一补”。继续推动重点行业化解过剩产能，创造条件推动企业兼并重组。规范住房租赁市场，着力化解房地产库存，商品房销售面积增长12%。支持企业市场化、法治化债转股，加大股权融资力度。积极化解政府债务风险，做好债务置换工作。落实降低实体经济企业成本各项政策，推动企业降本增效。抓住制约振兴发展的关键环节，着力补齐产业产品结构、金融发展、创新创业等方面的短板。

实施工业强基工程。全面推进质量、标准、品牌、信誉“四位一体”建设，努力争创国家级、省级知名品牌示范区。落实《中国制造2025鞍山行动纲要》，推动传统产业向中高端发展，促进缘泰化工、辽宁忠大高铁整车生产线等重点项目投产达产。壮大激光和电子信息、柔性输配电、智能机器人、新能源电池、节能环保装备等新兴产业规模，鼓励工业企业由生产型制造向服务型制造转变。支持鞍钢发展，促进鞍钢东部矿区项目实施，推进张家湾铁矿等采选一体化项目。鼓励地方企业参与鞍钢发展，促进地企深度合作。

促进现代服务业发展。着力推进服务业供给侧结构性改革，加快服务业市场开放，形成依靠生产性服务业助推服务输出的优势，推动生活性服务业走向精细化、高品质。促进兴隆大家庭等重点项目竣工，加快农产品交易中心建设。大力发展电子商务和物流业，促进大宗商品交易平台建设及线上线下融合发展。壮大金融服务业，实施“金融+”计划，以金融服务促进实体经济发展。积极争取投贷联动试点，大力发展股权投资基金、融资租赁、物流金融、供应链金融等新型金融业态，全面建设具有活力的金融生态环境。做大做强文化旅游产业，支持千山旅游集团发展壮大，筹建鞍山温泉旅游集团，提升温泉旅游、工业旅游、乡村旅游发展水平。加快发展文化体育、健康养老等现代服务业。

大力发展现代农业和县域经济。深入推进农业供给侧结构性改革，促进农民增收、农业增效、农村发展。强化农产品质量监管，增加绿色优质农产品供给，做强南果梨、食用菌等特色农业品牌，推进农产品深加工项目建设。粮食产量稳定在110.5万吨，设施蔬菜产量达到189万吨，调减玉米种植面积3.5万亩。加强农田水利基础设施建设。推进农作物秸秆综合利用。推动西柳、南台等专业市场向大宗商品采购平台转变，促进君子兰、观赏鱼等特色市场集群提档升级。推进以特色小镇为代表的新型城镇化建设，加快产业集聚，壮大特色优势产业，加速一二三产业融合。

（四）鼓励创新创业，汇集发展新动能。深入实施创新驱动战略，推动科技创新与制度创新、管理创新、商业模式创新、业态创新和文化创新相结合，培育发展新动能。

提升产业创新能力。积极融入沈大国家自主创新示范区。实施“创谷”计划，推进高新区创业基地建设。加快小巨人企业培育，实施科技型中小企业成长路线图计划，培育20家以上国家高新技术企业。积极促进与哈尔滨工业大学、中科院相关院所合作，推进高等院校产、学、研一体化建设。建设科技成果转化基地。举办科技成果推介洽谈会，实现企业技术需求与专家有效对接。

支持大众创业万众创新。加强创业平台建设，鼓励社会各界兴办众创、众包、众扶、众筹空间。制定实施“城市合伙人”行动计划，创办智谷空间、创客空间等一批新型孵化器，吸引聚集高端人才、资本等要素资源。实施楼宇经济行动计划，促进商务楼宇开发建设和改造升级，打造协作分享新经济。实施天使助力工程，促进创业者和投资者有效对接。提升科技大市场服务能力，积极建设科技金融超市。

集聚创新创业人才。通过落实高端人才引进政策、鼓励科研人员领办创办、支持现有企业孵化分离、推动科技成果就地转化等方式，鼓励科技人才、行业领军人才创新创业。充分发挥辽宁科技大学、鞍山师范学院创新引领作用，发现和培养青年科技人才、专门人才、创业人才，让人才成为全面振兴钢都的支撑和引领。

（五）不断改善民生，实现福祉新提升。把人民对美好生活的向往作为奋斗目标，努力为百姓提供更舒适的环境、更优质的服务，让老百姓的日子越过越好，让老百姓的烦心事越来越少。

全力实施脱贫攻坚。多渠道加大扶贫投入，着力解决贫困地区基础设施和公共服务瓶颈制约，建立健全产业到户到人的精准扶持机制。全年争取扶贫信贷风险基金达到5000万元以上，对参加新农合扶贫对象个人缴费部分给予全额资助，为贫困人口购买补充医疗保险，确保完成1.2万贫困人口脱贫、47个贫困村销号，岫岩摘掉省级重点贫困县的帽子。

做好就业和社会保障工作。落实就业促进和就业援助计划，全年城镇新增就业3.9万人。加强社会保险扩面征缴和基金收支管理，确保养老金按时足额发放。做好对企业困难职工、低收入人群和困难家庭的帮扶工作，保障城乡贫困群众的基本生活。基本建成鞍山市养老院，改造农村敬老院15所，推广医养结合服务模式，改善养老服务质量。推进保障性安居工程建设，实施棚户区改造1.8万套，基本建成1.2万套。

着力建设宜居城市。实施创建全国文明城市推进计划，让全市人民在共建共享中享有更多的获得感。推进园林大道等一批城市道路改造工程，加快建设城市停车场，缓解百姓停车难。新建过街天桥2处，在市管19条主干道新建盲道25.7公里。建成新殡仪馆。实施城市老旧小区综合整治改造工程，推进供水、供气、供热等地下管网提标改造，在已拆扒小锅炉原址上新建15个健身休闲街心游园。建设智慧城市，推进大数据云计算中心、城市智能运营中心等项目。实施城市绿化、亮化、净化、美化工程，普遍推行垃圾分类制度。开展全域农村环境综合整治，促进美丽示范村提档升级，打造城市后花园。加强生态环境保护，制定和实施《大鞍山生态区建设方案》，加大大气、水污染治理力度。推进冬季清洁取暖，拆除小锅炉251台。淘汰黄标车及老旧车辆9476辆。实施万水河、运粮河综合治理等17个碧水工程。人工造林2.9万亩。开工建设生活垃圾焚烧发电项目和城市污泥处理中心。

全面发展社会事业。加快推进农村薄弱学校改造工程和义务教育均衡发展示范县区创建工作，做好“双元”培育技术技能人才改革试点工作。深化“三医联动”改革，推进分级诊疗制度，加强社区卫生服务建设，提高公共卫生和基本医疗服务水平。建成市中心医院住院医师培训大楼和中国医大鞍山医院病房大楼。全面落实两孩政策。实施文化惠民工程，广泛开展全民健身运动。落实《公共文化服务保障法》，加快公共文化设施建设，实现档案馆、科技馆、青少年活动中心、妇女儿童活动中心、英模馆、群众艺术馆等项目主体完工，重新启动人才大厦、“城市之门”等项目建设。继续做好双拥模范城创建工作。

加强和创新社会治理。积极推进网格化社会治理和城市管理体系创新，提升基层社会治理科学化、法治化水平。建立完善社区公共服务事项准入制度和分层协商制度，开展城乡社区协商示范点建设。深入推进“七五”普法工作，建立法律服务平台。加强食品安全监管，建设食品放心城市。强化立体化社会治安防控体系建设，严格落实安全生产和信访稳定工作责任，扎实做好民族宗教工作，努力建设更高水平的平安鞍山。

进一步做好广播电视、新闻出版、妇女儿童、老龄、残联、外事、侨务、对台、统计、接待、档案、编制、气象、地震、人防、民兵预备役等工作。

（六）加强自身建设，树立政府新形象。只有真抓实干，全面振兴钢都的蓝图才能变为现实。我们要全面加强政府自身建设，强化责任担当，激励干事创业，努力打造人民满意的政府。

坚持廉洁从政，强化政府系统党风廉政建设。坚决落实全面从严治党要求，严明政治纪律和政治规矩，严格落实“一岗双责”责任制。强化重点领域、重点环节监管，扎紧制度笼子，坚决纠正损害群众利益行为，从源头上预防腐败。把落实中央八项规定精神作为一项铁的纪律，持之以恒抓好作风建设。坚持正向激励，把严格要求与关心关爱干部结合起来，让广大干部既遵规守纪，又心情舒畅，积极作为，建功立业。

坚持依法行政，把法治理念落实到政府工作全领域、全过程。自觉接受人大及其常委会的法律监督和政协的民主监督。依法全面履行政府职能，严格执行重大行政决策程序，落实行政执法责任制。深入推进政务公开，不断完善行政权力制约和监督机制，确保依规依纪依法用权。

坚持务实勤政，增强政府公信力和执行力。牢固树立正确的政绩观，以担当负责的态度解决好发展中问题和历史遗留问题，以政府诚信带动社会诚信。发扬求真务实、真抓实干的作风，全力推进全面振兴钢都首批“1+13”重大专项，做到高起点谋事、高效率做事、高质量成事。完善常态化督查机制，强化对重要决策、重点工作和重大项目的跟踪督办，推动各项决策部署落到实处。

各位代表，奋斗成就梦想，实干赢得未来。全面振兴钢都、再创鞍山辉煌的历史使命已经落在我们的肩上，让我们更加紧密地团结在以习近平同志为核心的党中央周围，在省委、省政府和市委的正确领导下，坚定信心、振奋精神、奋力拼搏，努力开创全面振兴钢都的新局面，以优异成绩迎接党的十九大胜利召开！
